# A history of childhood trauma is associated with slower improvement rates: Findings from a one-year follow-up study of patients with a first-episode psychosis

**DOI:** 10.1186/s12888-016-0827-4

**Published:** 2016-05-04

**Authors:** Monica Aas, Ole A. Andreassen, Sofie R. Aminoff, Ann Færden, Kristin L. Romm, Ragnar Nesvåg, Akiah O. Berg, Carmen Simonsen, Ingrid Agartz, Ingrid Melle

**Affiliations:** Division of Mental Health and Addiction, Institute of Clinical Medicine, NORMENT K.G Jebsen Centre for Psychosis Research, TOP study group, University of Oslo and Oslo University Hospital, Bygg 49, Ullevål sykehus, PO Box 4956, Nydalen, 0424 Oslo Norway; Department of Genetics, Environment and Mental Health, Norwegian Institute of Public Health, Oslo, Norway; Department of Psychiatric Research, Diakonhjemmet Hospital, Oslo, Norway; Department of Specialized Inpatient Treatment, Division of Mental Health Services, Akershus University Hospital, Oslo, Norway

## Abstract

**Background:**

The aim of this study was to investigate whether childhood trauma was associated with more severe clinical features in patients with first-episode psychosis, both at the initial assessment and after one year.

**Methods:**

Ninety-six patients with a first-episode of a DSM-IV diagnosis of psychosis, in addition to 264 healthy controls from the same catchment area, were recruited to the TOP NORMENT study. A history of childhood trauma was obtained using the Childhood Trauma Questionnaire (CTQ). Function and symptom severity were measured using the Global Assessment of Functioning (GAF) Scale divided into function (GAF-F) and symptoms (GAF-S), the Positive and Negative Syndrome Scale (PANSS) and the Young Mania Rating Scale (YMRS). All clinical assessments were completed at two time points: At an initial assessment within the first year of initiating treatment for psychosis and after one year.

**Results:**

Childhood trauma was associated with significantly reduced global functioning and more severe clinical symptoms at both baseline and follow-up, whereas emotional neglect was associated with a significantly reduced improvement rate for global functioning (GAF-F) over the follow-up period.

**Conclusion:**

Our data indicate that patients with first-episode psychosis who report a history of childhood trauma constitute a subgroup characterized by more severe clinical features over the first year of treatment, as well as slower improvement rates.

**Electronic supplementary material:**

The online version of this article (doi:10.1186/s12888-016-0827-4) contains supplementary material, which is available to authorized users.

## Background

Childhood trauma is a well-documented potential risk factor for psychosis. A recent large-scale meta-analysis, including *n* = 45,441 individuals, concluded that childhood trauma is strongly linked to an increased risk of developing a psychotic disorder, with an overall effect of OR = 2.78 (95 % CI = 2.34–3.31) [1]. A recent study of individuals at a high risk of psychosis also showed that a history of childhood sexual abuse increased the transition rate to psychosis from two to four times [2], while others have found that childhood trauma increases the likelihood of a specific admixture of affective-, anxiety- and psychotic symptoms that cut across traditional diagnostic boundaries of mood disorders and schizophrenia [3]. Thus, there seems to be reasonably consistent evidence that childhood trauma is associated with an increased risk of developing a severe mental disorder in adulthood across clinical features, including positive symptoms, anxiety and suicidal behaviours.

Childhood physical- and sexual abuse are by far the most studied subtypes of childhood trauma in relation to severe mental illness, with fewer studies investigating associations with emotional abuse and neglect [4]. Including emotional abuse and neglect are important, as recent studies indicate these as important aspects of childhood trauma in both schizophrenia and bipolar disorders [5–7].

There are several possible illness trajectories for patients experiencing a first-episode psychosis. Many develop a serious long-term illness, although there is a considerable variation in functional levels over time and between patients groups [8]. To be able to better predict the development of a severe long-term illness has major clinical implications, since this may help to facilitate potentially effective tertiary prevention strategies. Thus far, there has not been enough knowledge to allow reliable predictions, but a recent review article on risk factors for developing a severe course of schizophrenia illness indicates that poor premorbid function, together with a long duration of untreated psychosis (DUP), are important factors [9]. Individuals at a high risk of psychosis who report a history of childhood trauma score lower on premorbid adjustment compared to individuals without childhood trauma [10]. A history of childhood trauma could therefore be a factor associated with a poor premorbid function. It could also be that individuals with childhood adverse events are later in seeking help for psychotic symptoms, hence potentially creating a longer DUP. To the best of our knowledge, our study is the first of its kind to investigate these aspects of childhood trauma in a longitudinal study design. Because females tend to report a higher prevalence of trauma than males [7], as well as different associations to trauma [11, 12], we will also investigate correlates of gender. Lastly, as patients with schizophrenia spectrum disorders report higher incidences of childhood trauma than patients with affective psychoses [11], we will also investigate potential diagnosis correlates.

Our hypotheses are as follows: 1) Patients with first-episode psychosis will report significantly more childhood trauma experiences compared to controls from the same catchment area; 2) A history of childhood trauma will be associated with reduced global functioning and a more severe symptom profile, both at the initial assessment and after one year in treatment; 3) A history of childhood trauma will be associated with a slower improvement rate over time, which is indicative of a more severe course and outcome in the traumatized patients; and 4) A long DUP and poor premorbid function will mediate the relationship between childhood trauma and a slower improvement rate over time.

## Methods

### Participants

The participants were recruited from psychiatric units (outpatient and inpatient) in four major hospitals in Oslo as part of the larger Thematically Organized Psychosis (TOP) NORMENT Research Study. Patients were included from November 2007 until August 2012. Participants were recruited within the first year of being treated for a psychotic episode (*N* = 163). Of the 163 patients participating at baseline, 96 agreed to meet for follow-up assessment. There were no statistically significant baseline differences in demographic or clinical variables between patients who participated and those who did not participate at follow-up (data not shown).

Of the 96 participants who completed follow-up assessments forty had a schizophrenia spectrum disorder [30 with schizophrenia, three with schizophreniform disorder, seven with schizoaffective disorder and 17 with other types of psychosis] and 39 had a psychotic affective disorder [33 with bipolar I, two with bipolar II, two with bipolar NOS and two with a major depressive disorder with psychotic features (one-year follow-up diagnoses)]. A history of psychosis in psychotic affective disorder patients was based on information retrieved from the SCID interview. Eighty-one (84 %) of the patients were taking medication at the baseline assessment, 73 (76 %) were taking at least one type of antipsychotic medication, 25 (24 %) used antidepressant medication and 22 (23 %) of the patients were taking mood stabilizers. The mean age of the patients at baseline was 27.4 ± 8.3 years and *n* = 54 (56 %) were males. Moreover, a healthy control group of 264 individuals without any Axis 1 disorders or ongoing illicit drug abuse were recruited from the same geographical areas as the patients (Controls’ age mean ± SD: 30.1 ± 7.9; 56 % males). All controls had complete data on childhood trauma experiences. To help counteract the effects of socio-economic differences between different parts of the city, controls were also randomly recruited from the same city areas as the patients. The exclusion criteria for all groups were an unstable or uncontrolled medical condition that interfered with brain function, and an age outside the range of 18–65 years.

### Clinical assessment

Trained psychiatrists, physicians and clinical psychologists carried out the clinical assessment, and a diagnosis was based on the Structured Clinical Interview for DSM-IV Axis I disorders (SCID-I). The diagnostic reliability was found to be satisfactory [13], with an overall agreement for DSM-IV diagnostic categories of 82 % and an overall κ of 0.77 (95 % CI: 0.60–0.94). All clinicians who performed the clinical assessments were trained and supervised by the UCLA training programme [14]. A recent five-factor model for the PANSS was conducted [15] and used in this study. Symptom severity and function were rated separately using a split version of the Global Assessment of Functioning Scale (GAF; [16]. Current manic symptoms were measured with the Young Mania Rating Scale (YMRS) [17]. Furthermore, the inter-rater reliability of the symptom assessments in the TOP study have been shown to be good, with an Intraclass Coefficient (ICC) for PANSS symptoms of 0.82, a GAF-F of 0.85 and a GAF-S of 86 [18, 19].

Medication at the time of testing was determined through clinical interview and medical charts, whereas the untreated psychosis DUP was defined as the time from the first psychotic experience for more than seven days until treatment for a psychotic illness. Reliable data on DUP was available for *n* = 83 (87 %) of the patients, with a median (min-max) of 14 (0–1040 weeks). First-episode psychosis was defined as patients within the first year of treatment for a schizophrenia spectrum disorder or psychotic affective disorder. Premorbid functioning was assessed by use of the Premorbid Adjustment Scale (PAS) [20], and we focused here on premorbid function in childhood, including social and academic functioning. High scores on the PAS signify a poor social and cognitive functioning in childhood.

### Childhood Trauma Questionnaire (CTQ)

Traumatic events in childhood were rated using a Norwegian version of the Childhood Trauma Questionnaire (CTQ) (see [21, 22]. This is a self-report questionnaire with 28 items [22], yielding scores on five subscales of trauma: emotional abuse (EA), physical abuse (PA), sexual abuse (SA), physical neglect (PN) and emotional neglect (EN), as well as a total score; for more detailed information, see [21, 23]. Each subscale is measured with five items rated on a 5-point Likert scale from 1 (never true) through 5 (very often true). Childhood trauma data were analysed for subscales, together with an overall test of childhood trauma, with all the subscales added together.

### Statistical analyses

Data were analysed using the Predictive Analytic Software (PASW), Version 21 (formerly SPSS Statistics). Because the childhood trauma data and several clinical variables (PANSS and YMRS) were not normally distributed, a non-parametric test (Spearman’s correlation) was performed to investigate associations between childhood trauma as a continuous variable and the clinical features in first-episode psychosis at both baseline and the 12-month follow-up. A paired sample *t*-test was used to compare normally distributed clinical data (GAF-F and GAF-S scores) at baseline with the one-year follow-up. A Mann–Whitney test was used to investigate the differences in childhood trauma scores in patients compared to controls. For the CTQ and the clinical data that were not normally distributed, the data were log transformed before being added into the following parametric analysis (this included CTQ, PANSS items, DUP and a premorbid adjustment score in childhood from the PAS; the CTQ and the PAS were still skewed after transformation, and were therefore dichotomized into a ≤ and ˃ median score approach). Repeated measures of Analysis of variance (ANOVA) were performed to investigate the interaction effects between time (first-episode psychosis to the one-year follow-up, within-subjects variable) and childhood trauma (divided into ≥ median, and > median score, between-subjects factor) on clinical measures. The effect size was measured by the < eta > 2, while the small, moderate to large effect size was defined as < eta > 2 ANOVA 0.01, 0.06, 0.14 [24]. In the tables, “Bold” represent *p*-values <0.05 and (*) represent *p*-values  <0.01.

## Results

### Childhood trauma in patients and in controls

Patients with a first-episode psychosis reported significantly more childhood trauma experiences across all subtypes of childhood trauma than healthy controls from the same catchment area (*p* < 0.001) (see Table 1). However, as we can see in Table 1, the childhood trauma scores were skewed to the left in both groups, with a median score of 5 (i.e. low) for physical and sexual abuse in both patients and controls. However, a subgroup of patients had more extreme scores, leading to clear differences compared to controls on the non-parametric tests. Furthermore, females reported a higher prevalence of sexual and emotional abuse than males (*z* = −2.91, *p* = 0.004 and *z* = 2.70, *p* = 0.007, respectively), though with no gender differences observed for the childhood total trauma score (*z* = −1.37, *p* = 1.69; see Additional file 1: Table S1). Similar findings were also observed in the control sample (data not shown). Patients with a schizophrenia spectrum diagnosis reported higher levels of physical abuse than patients with a psychotic affective disorder (*z* = −2.23, *p* = 0.026), as well as more physical neglect (*z* = −2.00, *p* = 0.045) and differences at a trend level for higher childhood total trauma score (*z* = −1.66, *p* = 0.098), see Additional file 1: Table S2).

**Table 1:** Demographic characteristics and childhood trauma in patients and healthy controls

	Patients *N* = 96	Controls *N* = 26	Statistics
Age (mean ± SD)	27.4 ± 8.3	30.07 ± 7.9	*f* = 0.81, t = −2.26, *p* = 0.02
Gender M/F	54/42	148/116	*X* ^2^ = 0.001, df = 1, *p* = 0.97
Childhood trauma (CTQ)^a^			
Total score, median (min-max)	41 (25–77)	29 (25–53)	*z* = −9.40, *p *< 0.001
Physical abuse, median (min-max)	5 (5–19)	5 (5–11)	*z* = −8.74, *p *<0.001
Sexual abuse, median (min-max)	5 (5–21)	5 (5–14)	*z* = −6.65, *p *< 0.001
Emotional abuse, median (min-max)	10 (5–24)	5 (5–19)	*z* = −9.14, *p *< 0.001
Emotional neglect, median (min-max)	11 (5–24)	7 (5–25)	*z* = −8.00, *p *< 0.001
Physical neglect, median (min-max)	7 (5–17)	5 (5–13)	*z* = −8.04, *p *< 0.001

^a^ Mann-Whitney test

### Clinical features at baseline for first-episode psychosis and after one-year follow-up

At baseline, the total sample of patients had moderately high symptom levels, with a significant improvement from baseline to the one-year follow-up for the PANSS positive-, negative-, excited- and depressive factors. On average, both GAF functions and symptoms (GAF-F and GAF-S) improved 10 points from baseline to the one-year follow-up assessment (see Table 2).

**Table 2:** Clinical characteristics of patients at baseline and at 1-year follow-up

	Baseline *n* = 96	1-year follow-up *n* = 96	Statistics
Diagnosis n (%)			
Schizophrenia spectrum	55 (57)	57 (59	
Bipolar disorders	41 (43)	39 (41)	
GAF-S (mean ± SD)	47.5 ± 12.8	57.3 ± 16.7	*t* = 6.61, *p* < 0.001
GAF-F (mean ± SD)	47.3 ± 11.9	57.0 ± 15.8	*t* = −6.01, *p* < 0.001
YMRS median (min-max) ^a^	4 (0–28)	2 (0–16)	*Z* = −4.88, *p* < 0.001
PANSS 5 factor			
Positive factor, median (min-max), median (min-max)	9 (4–23)	6 (4–22)	*Z* = −5.12, *p* < 0.001
Negative factor, median (min-max)	10 (6–27)	8 (6–22)	*Z* = −3.39, *p* < 0.001
Disorganized/concrete factor, median (min-max)	5 (3–14)	4 (3–13)	*Z* = −1.64, *p* = 0.10
Excited factor, median (min-max)	6 (4–13)	4 (4–13)	*Z* = −3.45, *p* < 0.001
Depressed factor, median (min-max)	9 (3–15)	7 (3–16)	*Z* = −5.22, *p* < 0.001
Medication			
At least one type of Antipsychotic medication, yes n (%)	73 (76 %)	74 (77)	*X* ^2^ = 0.97, *p* = 0.33
DUP (weeks)	104.7 ± 192.6		
PAS score in childhood	0.23 ± 0.19		

^a^ Wilcoxon signed-rank test; *YMRS* Young Mania Rating Scale, *DUP* duration of untreated psychosis, *PAS* premorbid adjustment scale

### Childhood trauma and clinical features in first-episode psychosis

The childhood trauma total score was associated with reduced global functioning as measured by the GAF-F and by more severe clinical symptoms, particularly affective symptoms (PANSS depressive factor), at both the initial assessment and the one-year follow-up (see Table 3). When dividing trauma into subtypes, the strongest association for the severity of clinical features was observed for childhood physical- and emotional abuse, both at the initial assessment and the one-year follow-up. The strongest overall relationship between any childhood trauma and symptom dimension was found for depressive symptoms (PANSS depressive factor), with a consistent statistically significant association to the childhood trauma total score, and to all subtypes of trauma at both time points.

**Table 3:** The association between childhood trauma and clinical symptoms at baseline and at 1 year follow-up

	Physical abuse		Sexual abuse		Emotional abuse		Emotional neglect		Physical neglect		All trauma	
	Baseline	1 year	Baseline	1 year	Baseline	1 year	Baseline	1 year	Baseline	1 year	Baseline	1 year
GAF-S	***r*** = −0.42*	***r*** = −0.25*	*r* = −0.15	*r* = −0.07	***r*** = −0.27*	***r*** = −0.26*	***r*** = −0.27*	***r*** = −0.31*	*r* = −0.20	***r*** = −0.28*	***r*** = −0.31*	***r*** = −0.29*
GAF-F	***r*** = −0.28*	***r*** = 0.30*	*r* = 0.07	*r* = −0.17	*r* = −0.12	***r*** = −0.30*	*r* = −0.17	***r*** = −0.38*	*r* = −0.14	***r*** = 0.35*	*r* = −0.14	***r*** = −0.40*
YMRS	***r*** = 0.34*	*r* = 0.18	*r* = 0.19	*r* = 0.16	***r*** = 0.30*	***r*** = 0.35*	***r*** = 0.27*	***r*** = 0.32*	***r*** **= 0.25**	***r*** = 0.29*	***r*** = 0.34*	***r*** = 0.33*
PANSS 5 Factor (F)												
Positive F	***r*** = 0.27*	*r* = 0.16	*r* = 0.02	*r* = −0.17	*r* = 0.13	*r* = 0.15	***r*** = 0.25*	*r* = 0.19	*r* = 0.11	***r*** **= 0.23**	*r* = 0.16	*r* = 0.13
Negative F	***r*** **=** 0.13	*r* = 0.18	*r* = −0.05	*r* = −0.04	*r* = 0.05	*r* = 0.13	*r* = 0.08	*r* = 0.16	*r* = 0.06	***r*** **= 0.23**	*r* = 0.05	*r* = 0.16
Disorganized/concrete F	***r*** **= 0.24**	***r*** = 0.29*	*r* = 0.04	*r* = −0.03	*r* = 0.07	*r* = 0.17	*r* = 0.05	***r*** **= 0.22**	*r* = 0.06	***r*** **= 0.20**	*r* = 0.08	***r*** **= 0.21**
Excited F	***r*** **= 0.21**	***r*** = 0.28*	***r*** = 0.32*	***r*** = 0.26*	***r*** = 0.29*	***r*** = 0.35*	*r* = 0.16	***r*** **= 0.21**	*r* = 0.09	*r* = 0.18	*r* = 0.23	***r*** = 0.30*
Depressed F	***r*** = 0.35*	r = 0.27*	***r*** = 0.28*	***r*** **= 0.24**	***r*** = 0.32*	***r*** = 0.33*	***r*** = 0.31*	***r*** = 0.32*	***r*** **= 0.25**	***r*** = 0.37*	***r*** = 0.38*	***r*** = 0.38*

Spearman’s correlation, “Bold” represent *p*-values ≤0.05 and star (*) represent *p*-values of *p* ≤ 0.01)

### Clinical features and change from initial assessment to one-year follow-up

**Fig. 1:**
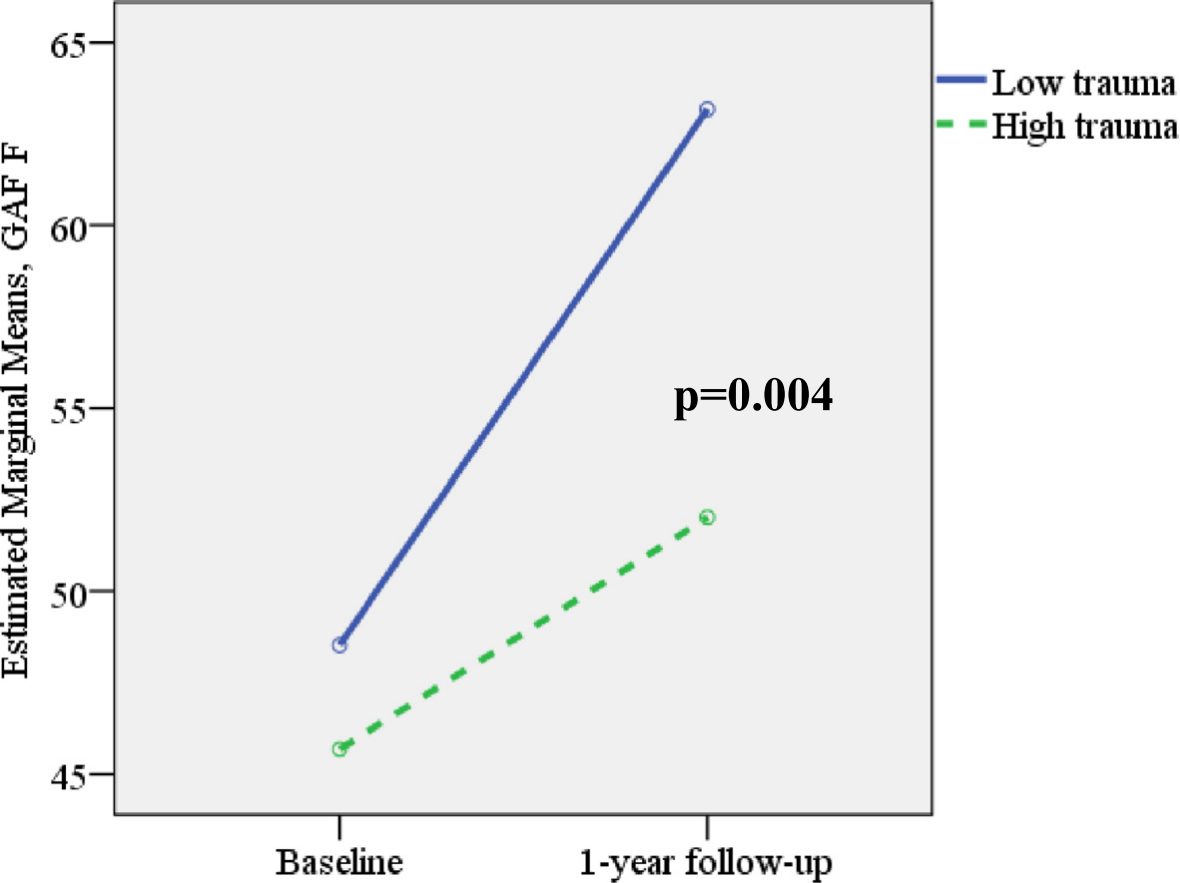
GAF function at baseline and 1-year follow-up and childhood emotional neglect high vs low scores - interaction effect over time. Figure 1 shows that emotional neglect is associated with lower GAF scores at baseline and after one year follow-up. *Repeated measures of ANOVA: Interaction time x trauma: f = 8.59, p = 0.004; effect size eta^2 = 0.09; N = 50 low trauma; N = 44 high trauma; GAF function baseline score low trauma: 48.4 ± 11.8, high trauma baseline: 45.6 ± 11.1: GAF function 1 year follow-up low trauma 62.8 ± 11.9, high trauma 51.0 ± 11.2*

Having a high childhood trauma total score (above the median score) was associated with less improvement over time in global function as measured by a lower GAF-F (repeated measures ANOVAs interaction, time [baseline assessment and follow-up] x childhood trauma total score: f = 6.56, *p* = 0.01, effect size < eta > 2 = 0.07). When dividing into different subtypes of trauma, the most significantly reduced slope of improvement over time was found for emotional neglect (interaction term, time [the GAF-F baseline and follow-up] x emotional neglect: f = 8.59, *p* = 0.004; effect size < eta > 2 = 0.09, see Fig. 1).

A similar finding was observed for physical neglect and GAF-F (f = 5.55, *p* = 0.02, effect size < eta > 2 = 0.06) and for sexual abuse on GAF-F (f = 5.13, *p* = 0.03, effect size < eta > 2 = 0.05). High physical abuse exhibited a significant association with a reduced functioning at both time points, with no interaction effects over time.

Patients reporting high scores on the childhood trauma total score had a significantly poorer premorbid adjustment in childhood (Spearman’s correlation: *r* = 0.27, *p* = 0.01), and a significantly longer duration of untreated psychosis (DUP) compared to patients without trauma (Spearman’s correlation: *r* = 0.36, *p* = 0.001). The DUP was also significantly correlated with a reduced ability to function, and with more global symptoms as measured by GAF-F and GAF-S at the 12-month follow-up (Spearman’s correlation: *r* = 0.34 and *r* = 0.47, and *p* = 0.002 to *p* < 0.001, respectively). Low premorbid function in childhood as measured by the PAS was associated with lower scores on GAF-F at the 12-month follow-up. Patients with a psychotic affective disorder had significantly better functioning (GAF-F) after 12 months, and less severe symptoms at baseline (GAF-S) compared to the patients with a schizophrenia spectrum diagnosis (data available on request). No gender differences were observed for GAF-F or GAF-S scores at baseline or at the 12-month follow-up. When DUP, premorbid function, gender and diagnosis were added as covariates into the repeated measures ANCOVA, emotional neglect was still associated with a slower improvement slope for global function (measured by GAF- F; f = 6.22, *p* = 0.01, effect size < eta > 2 = 0.08). No significant effect ofv DUP or poor premorbid function, gender or diagnosis was found in the final model (f = 0.25 ~ 3.99, *p* = 0.62 ~ 0.05, see Table 4).

**Table 4:** Repeated measure ANCOVA, emotional neglect and global function over time (GAF F at baseline and GAF F after 12 months)

	*f*	*DF*	*p*	*Effect size* < eta > 2
Gender M/F	3.99	1	0.05	0.05
Diagnosis	3.19	1	0.08	0.04
PAS	0.25	1	0.62	<0.01
DUP	0.49	1	0.49	0.01
Emotional neglect score	6.22	1	**0.015**	0.08

“Bold” represent *p*-value <0.05

## Discussion

In the current study, patients with first-episode psychosis reported more childhood trauma experiences than the healthy control group. Additionally, a history of childhood trauma in patients was related to poorer functioning and more severe affective symptoms at baseline and at the one-year follow up, with a slower improvement rate over time. Effect sizes were moderate. These features appeared to be independent of both poor premorbid social and academic functioning in childhood, and of the duration of untreated psychosis (DUP), both of which are known factors associated with a poor outcome in this patient group.

Here, we replicated former reports of more prevalent childhood trauma experiences in first-episode psychosis compared to healthy controls [6, 25, 26]. Associations between high rates of trauma and clinical features were especially pronounced for global function- and symptom measures (GAF-F and – GAF-S scores), as well as for affective symptoms as measured by the PANSS depressive factor. At both time points, the strongest cross-sectional associations between psychopathological measures and subtypes of childhood trauma were observed for different types of abuse (compared to neglect).

It is of particular interest that the strongest associations of childhood trauma were related to depressive symptoms, both at the initial- and the follow-up assessment. Depression is common in first-episode psychosis [27], with an increased risk of suicide during the early phases of psychotic illness, in addition to a reduced quality of life [28, 29]. Our findings of more severe current clinical symptoms in patients with high levels of trauma also have a concrete, functional clinical relevance, as indicated by a reduced global function in this subgroup of patients. These findings were found independently of gender and diagnosis. As a result, it is most likely that a history of childhood trauma is a general risk factor for adverse mental health outcomes, including psychosis, thus cutting across diagnostic categories. Without a comparison to people experiencing different symptom constellations (e.g. non-psychotic depression or anxiety disorders), it is difficult to be precise about the specific role of trauma in the ontogeny of psychosis.

Interestingly, although the neglect items had lower cross-sectional effects on global function and symptoms (GAF-F and GAF-S scores), they had a significant effect on the slope of symptom improvement over the follow-up period. One possible mechanism could be that compromised emotion regulation strategies in individuals subjected to emotional neglect [30] could be followed by reduced emotion regulation skills, which would make these individuals less capable of dealing with the stress of experiencing psychotic symptoms.

We did not find validation for a long DUP, and poor premorbid function would help mediate the relationship between childhood trauma and a slower improvement rate over time. In fact, when all items were added into a multivariate model at the same time, only childhood trauma remained significant. The relationship between a poor premorbid functioning in childhood and childhood trauma is not straightforward: It has been proposed that having a poor cognitive functioning in childhood increases the risk of experiencing childhood trauma [31]. It is also well-known that both acute- and chronically high levels of stress exposure, particularly chronic stress in early life, have a more severe effect on brain development and plasticity compared to short-term stress and chronic stress later in life [32–34], thereby potentially influencing neurodevelopmental development and premorbid academic function in childhood. Early trauma could also affect a person’s ability to relate to others, and hence have a negative impact on premorbid social function. Our findings also indicate childhood trauma as a potential underlying risk factor for a long duration of untreated psychosis (DUP). It could be that patients with a history of childhood trauma are more sceptical or have a reduced trust towards others, and are therefore more reluctant to seek help when starting to experience psychotic symptoms.

Our results - based on measures at two time-points - suggest that childhood trauma is associated with a more severe psychotic illness, both during the first treatment and after one year. It is possible that worse abuse histories may confer a greater of risk of relapse, readmission, treatment resistance or other varied outcomes. Based on our results, it seems possible to conclude that recovery outcomes are worse in the first 12 months of FEP for people with childhood abuse, but only continued follow-up and outcome assessment will determine whether these effects persist over longer periods. This relationship between childhood trauma and the development of symptomatology and functioning in a psychosis population with a short treatment history has a number of clinical implications: The exposure to childhood trauma should be routinely assessed in first-episode psychosis because it appears to be related to an increased risk for developing a more severe illness over time, and it has previously been reported that exposure to abuse is often not properly assessed in psychiatric treatment services [35]. Addressing the consequences of childhood trauma through the use of appropriate treatment approaches could help ameliorate the negative effect on course and outcome. As described in [2], this would include the use of different psychological techniques such as coping strategies, body awareness/mindfulness techniques and stress management. Based on the current findings, a focus on resolving the depressive symptomatology could also be attempted. Since psychotic disorders are among the main contributors to the global burden of disease, the identification of factors driving a more severe chronic illness over time is important. In fact, recent studies show that psychotic patients with a history of childhood trauma have a poorer quality of life and a reduced psychosocial functioning, compared to patients without trauma [36, 37]. A study by Lysaker et al. [38] also shows that more long-term psychotic patients with a history of childhood abuse have a poorer vocational functioning (measured by reduced work performance and hours spent working) thereby supporting the notion of reduced function over time in this group.

### Limitations

Childhood trauma was assessed retrospectively, and may have been subject to recall bias. However, the retrospective examination of childhood trauma in patients with psychosis has been found to be a valid and reliable source when collecting data in previous studies [39]. Furthermore, although GAF-F and GAF-S are often used to measure current functioning and symptom level, it is a global measure - and thus a crude measure. Our sample size was also modest (*N* = 96), and larger independent study samples are needed.

## Conclusions

Patients with first-episode psychoses reporting a history of childhood trauma constitute a subgroup of patients characterized with more severe clinical features in the first year of treatment, as well as slower improvement rates over time. In the current study, high levels of childhood trauma were also associated with a poorer premorbid adjustment and longer durations of untreated psychosis (DUP), two known risk factors for severe chronic psychosis [9]. When combined in a multivariate analysis, childhood trauma had an independent contribution to a reduced functional improvement over time, whereas premorbid adjustment and DUP no longer had a significant association to change over time. Hence, our data indicate a history of childhood trauma as a potentially underlying factor behind an increased DUP and a poorer premorbid function, and eventually a more severe chronic illness. Our findings highlight the importance of assessing trauma experiences in patients with first-episode psychosis and treatment targets focusing on overcoming these challenges.

### Ethics approval and consent to participate

The Regional Committee for Medical Research Ethics South Eastern Norway and the Norwegian Data Inspectorate approved the study, and all participants gave their written informed consent.

### Consent for publication

Not applicable.

### Availability of data and materials

No competing interests. Data available on request.

## Additional file

Additional file 1: Table S1. Childhood trauma prevalence divided into gender. Additional file 1: Table S1 shows the prevalence of childhood trauma divided into gender. Females report more sexual and emotional abuse than males. Table S2. Childhood trauma prevalence divided into schizophrenia and affective psychoses. Additional file 1: Table S2 shows the prevalence of childhood trauma divided into schizophrenia and affective psychoses. Patients with schizophrenia report more often physical abuse and physical neglect compared to patients with an affective psychoses. (DOCX 17 kb)

## Abbreviations

CTQ: childhood trauma questionnaire
DSM-IV: Diagnostic and Statistical Manual of Mental Disorders, 4th. Edition.
DUP: Duration of untreated psychosis
EA: emotional abuse
EN: emotional neglect
GAF-F: Global Assessment of Functioning- Function
GAF-S: Global Assessment of Functioning- Symptoms
NORMENT: Norwegian Centre for Mental Disorders Research
PA: physical abuse
PANSS: positive and negative syndrome scale
PAS: premorbid adjustment scale
PN: physical neglect
SA: sexual abuse
SCID: structured clinical interview for DSM
TOP: thematically organized psychosis
UCLA: University of California, Los Angeles
YMRS: young mania rating scale
