# Supplementary material for: A history of childhood trauma is associated with slower improvement rates: Findings from a one-year follow-up study of patients with a first-episode psychosis
Source: BMC Psychiatry. 2016 May 4;16:126. doi: 10.1186/s12888-016-0827-4 (PMC4855869; doi:10.1186/s12888-016-0827-4)
Supplement: Additional file 1: Table S1. — Childhood trauma prevalence divided into gender. Additional file 1: Table S1 shows the prevalence of childhood trauma divided into gender. Females report more sexual and emotional abuse than males. Table S2. Childhood trauma prevalence divided into schizophrenia and affective psychoses. Additional file 1: Table S2 shows the prevalence of childhood trauma divided into schizophrenia and affective psychoses. Patients with schizophrenia report more often physical abuse and physical neglect compared to patients with an affective psychoses. (DOCX 17 kb) [file 12888_2016_827_MOESM1_ESM.docx]

**Supplementary material**

**Table S1:** Childhood trauma prevalence divided into gender

|  |  | **Males Females Statistics**  **N= 54 N=42** | | |  | |
| --- | --- | --- | --- | --- | --- | --- |
| Childhood trauma (CTQ)^a^ | |  |  |  | |  |
| Total score, median (min-max) | | 38.5 (25-71) | 43 (25-77) | z=-1.37, p=0.17 | |  |
| Physical abuse, median (min-max) | | 5 (5-17) | 5 (5-19) | z=-0.98 p=0.33 | |  |
| Sexual abuse, median (min-max) | | 5 (5-21) | 5 (5-20) | z=-2.91, p=0.004 | |  |
| Emotional abuse, median (min-max) | | 9 (5-20) | 12 (5-24) | z=-2.70, p=0.007 | |  |
| Emotional neglect, median (min-max) | | 11 (5-24) | 11 (5-24) | z=-00.3, p=0.98 | |  |
| Physical neglect, median (min-max) | | 7 (5-17) | 8 (5-13) | z=-0.46, p=0.46 | |  |
|  | |  |  |  | |  |

*^a^=Mann-Whitney test.*

**Table S2:** Childhood trauma prevalence divided into schizophrenia and affective psychoses

|  |  | **Schizophrenia Affective psychoses Statistics**  **N=57 N=39** | | |  | |
| --- | --- | --- | --- | --- | --- | --- |
| Childhood trauma (CTQ)^a^ | |  |  |  | |  |
| Total score, median (min-max) | | 43 (25-77) | 39 (25-53) | z=-1.66, p=0.098 | |  |
| Physical abuse, median (min-max) | | 6 (5-18) | 5 (5-19) | z=-2.23, p=0.026 | |  |
| Sexual abuse, median (min-max) | | 5 (5-21) | 5 (5-23) | z=-0.59, p=0.55 | |  |
| Emotional abuse, median (min-max) | | 10 (5-23) | 9.5 (5-24) | z=-0.81, p=0.42 | |  |
| Emotional neglect, median (min-max) | | 11 (5-24) | 11 (5-24) | z=-1.22, p=0.22 | |  |
| Physical neglect, median (min-max) | | 8.5 (5-17) | 7 (5-13) | z=-2.00, p=0.045 | |  |
|  | |  |  |  | |  |

*^a^=Mann-Whitney test.*
